# A Systematic Review and Meta-Analysis of EEG, fMRI, and fNIRS Studies on the Psychological Impact of Nature on Well-Being

**DOI:** 10.3390/ijerph23030377

**Published:** 2026-03-17

**Authors:** Alexandra Daube, Yoshua E. Lima-Carmona, Diego Gabriel Hernández Solís, Jose L. Contreras-Vidal

**Affiliations:** 1 Noninvasive Brain–Machine Interface System Laboratory, IUCRC BRAIN Center, University of Houston, Houston, TX 77204, USA; ad2434@rwjms.rutgers.edu (A.D.); dherna97@cougarnet.uh.edu (D.G.H.S.); 2 Department of Neuroscience and Behavioral Biology, Emory University, Atlanta, GA 30322, USA; 3 IUCRC BRAIN Center, School of Engineering and Sciences, Tecnologico de Monterrey, Monterrey 64700, Mexico

**Keywords:** nature, EEG, fMRI, fNIRS, psychology, well-being, neural exposome

## Abstract

**Highlights:**

**Public health relevance—How does this work relate to a public health issue?**

- A total of 33 studies, focused on understanding the psychological and physiological impact of nature on well-being, were identified, including a total of 2101 participants.
- Nature exposure decreased negative emotions in healthy and clinical populations, suggesting a therapeutic benefit that may support policies aimed at protecting and restoring natural environments to promote well-being.

**Public health significance—Why is this work of significance to public health?**

- In total, 23 studies were conducted in non-Western settings, highlighting a significant demographic gap and the need to include more research from Western environments.
- Only 1.4% of the participants were children, highlighting the need for more research on nature’s psychological and physiological impact in pediatric populations.

**Public health implications—What are the key implications or messages for practitioners, policy makers and/or researchers in public health?**

- EEG studies should expand their scope to incorporate metrics like functional connectivity, while prioritizing the standardization of real-world data for study comparisons and the effective inclusion of AI.
- Future research should include different geographical and climate conditions as well as longitudinal data to assess the long-term impact of urban green spaces and natural environments on brain health and psychological well-being, while supporting the international neural exposome initiative.

**Abstract:**

Exposure to nature has been associated with benefits to human well-being, commonly evaluated using standardized psychological assessments and, more recently, neuroimaging modalities such as Electroencephalography (EEG), functional Magnetic Resonance Imaging (fMRI), and functional Near-Infrared Spectroscopy (fNIRS). This systematic review and meta-analysis addresses the following questions. (1) How is the impact of nature on well-being studied using psychological and neuroimaging modalities and what does it reveal? (2) What are the challenges and opportunities for the deployment of wearable neuroimaging modalities to understand the impact of nature on the brain’s health and well-being? A search on PubMed, IEEE Xplore, and ClinicalTrials.gov (March 2024) identified 33 studies combining neuroimaging and psychological assessments during exposure to real, virtual or imagined natural environments. Studies were analyzed by tasks, populations, neuroimaging modality, psychological assessment, and methodological quality. Most studies were conducted in Asia (*n* = 23 or 70%). Healthy participants were the dominant target population (70%). In total, 61% of the studies were conducted in natural settings, while 39% used visual imagery. EEG was the most common modality (82%). STAI (*n* = 8) and POMS (*n* = 8) were the most common psychological assessments. Only seven studies included clinical populations. Two separate meta-analyses of nine studies with explicit experimental and control groups revealed a significant positive effect of nature exposure on psychological outcomes (Hedges’ g = 0.30; *p* = 0.0021), and a larger effect on neurophysiological outcomes (Hedges’ g = 0.43; *p* = 0.0004), both with moderate-to-high heterogeneity. Overall, exposure to nature was associated with reductions in negative emotions in clinical populations. In contrast, healthy populations showed a more balanced psychological response, with nature exposure being associated with both increases in positive emotions and reductions in negative emotions. Notably, 88% of the studies presented methodological weaknesses, highlighting key opportunities for future neuroengineering research on the neural and psychological effects of nature exposure.

## 1. Introduction

For centuries, people of all cultures and disciplines have explored the relationship between nature and human well-being, with substantial evidence supporting nature’s influence on psychological health. Theories like Stress Reduction Theory (SRT; [1]) postulate that spending time in nature can reduce stress symptoms, foster positive emotions, and improve cognitive functions due to the innate connection of humans to the natural world. According to SRT, appreciation of nature scenes activates the parasympathetic nervous system [2], which predominates during periods of rest and governs restorative physiological processes, such as digestion [3]. Similarly, Attention Restoration Theory (ART; [4]) proposes that positive responses to nature allow the restoration of attention [5,6]. Consistent with these theoretical frameworks, empirical studies demonstrate that nature exposure positively influences multiple dimensions of well-being, from reductions in stress elicited by visual stimuli to increases in positive mood resulting from direct contact with natural environments [7,8,9,10]. Additionally, although physical immersion in nature appears to produce the strongest effects, simulated nature experiences, such as imagery or videos, have also been shown to improve aspects of well-being [9,11].

Growing interest in how nature influences the brain has driven research toward understanding its neurophysiological impact. This interest has recognized that genetics alone cannot account for health risk factors [12], and that environmental exposure plays a substantial role in shaping brain health [13]. This combination of external exposures with behavioral and endogenous factors, and their impact on brain health, is conceptualized as the “neural exposome” [12]. This emerging concept allows for the investigation of the impact of non-inheritable factors, such as the environment, on the brain and general health across the lifespan [14]. Within this framework, exposure to natural environments has been associated with positive impacts on people affected by chronic diseases, including cancer [12], cardiovascular conditions [15], and stress-related illnesses [16]. Additionally, interaction with nature directly impacts behavioral factors, as studies have shown that it improves sleep quality [17] and reduces stress [18]. These two behavioral factors directly influence brain health, as sleep is required for executive and cognitive function [19], and stress impacts overall brain health through Hypothalamic–Pituitary–Adrenal sensitization [20]. Therefore, investigating how nature-based interventions can be used to mitigate behavioral issues, such as stress and psychological conditions, has emerged as a growing body of research.

Historically, research on evoked responses by the perception of environmental stimuli relies primarily on subjective psychological assessments. These assessments have been widely used to evaluate outcomes such as stress, mood, anxiety, and attention. On the other hand, objective measurements, such as physiological indicators (heart rate variability, blood pressure, etc.), are a more recent development and represent essential complementary measures for quantifying the biological impact of nature [21]. However, among these indicators, neurophysiological mechanisms have not been fully considered due to the constraints associated with traditional neuroimaging modalities, which typically require stationary conditions and therefore restrict the ability to investigate brain mechanisms in naturalistic environments.

To quantify the effect of nature not only on subjective well-being, but also on brain health, quantitative biometrics provided by neuroimaging modalities must be included. Functional Magnetic Resonance Imaging (fMRI) has been implemented to address this need, as it offers an excellent spatial resolution compared to other neuroimaging modalities. However, it is not suitable for ecological studies in real-world settings due to practical restrictions forced by the scanner environment, which requires participants to remain stationary [22]. Advances in Mobile Brain–Body Imaging (MoBI) technologies now make it possible to study neural cognition and the behavior of individuals in complex real-world environments [23]. Among these technologies, Electroencephalography (EEG) has become a prominent modality in many clinical and research settings due to its noninvasive, reasonably inexpensive nature and high temporal resolution. EEG studies have shown variations in brain wave activity in the human brain and are widely used as a method to measure stress from recorded neural signals [24,25]. Because of EEG’s application in stress research, the modality has gained interest in emotion recognition and mental health research in the past 20 years [26]. While studies have used EEG for imagery-based investigations [26], mobile EEG allows for the investigation of other factors like the physiological impact of the natural or built environment [27]. One of the first studies to explore this relationship, using an automated EEG emotion classification software, concluded that the use of EEG to quantify health improvements would be beneficial, but also noted that there were limitations to emotion classifications, including the lack of storage of raw data [27]. Another study urged caution in interpreting EEG data, especially for studies performed in nature, due to their limited number and the inconsistencies in some of the results [28], probably due to physiological and non-physiological artifacts that contaminated the EEG recordings. In addition to EEG, another MoBI technology that has been employed in similar studies is functional Near-Infrared Spectroscopy (fNIRS). fNIRS has a higher tolerance for motion and portability, like some mobile EEG devices, but its lower spatial resolution and limited recording depth limit its applicability.

Given these methodological considerations, understanding the state of research over the past 20 years that uses neuroimaging combined with psychological assessments is of interest to identify knowledge gaps (e.g., neural mechanisms) and opportunities for developing nature-based interventions to improve brain health and well-being. The importance of these three modalities lies in the spectrum of their ability to record neurophysiological changes from different forms of nature or simulated stimuli, and the introduction of a new quantitative approach to studies that previously relied on qualitative data.

Studies employing psychological and neuroimaging modalities can potentially provide a shift in our understanding of the neurophysiological impact of nature on brain health and well-being. Such studies have considerably increased in popularity by exploring different natural stimuli and also using different neurological modalities [27,29,30]. However, despite this growing interest, the evidence remains fragmented across modalities. Moreover, methodological variability, limited ecological validity, and differences in study designs make it difficult to determine the extent to which nature exposure reliably influences neural processes.

Thus, the key objective of this systematic review is to analyze the state of research on the impact of nature on brain activity and mental well-being measured psychologically and neurophysiologically, and to clarify how these two domains relate. To address the gaps identified above, this review is guided by two central questions. (1) How is nature’s impact on well-being studied using psychological assessments in combination with neuroimaging modalities, and what does this integrated evidence reveal about its effects on brain activity and well-being? (2) What are the challenges and opportunities for deploying wearable neuroimaging tools to advance real-world investigations into nature’s impact on brain health?

## 2. Materials and Methods

**Figure 1:**
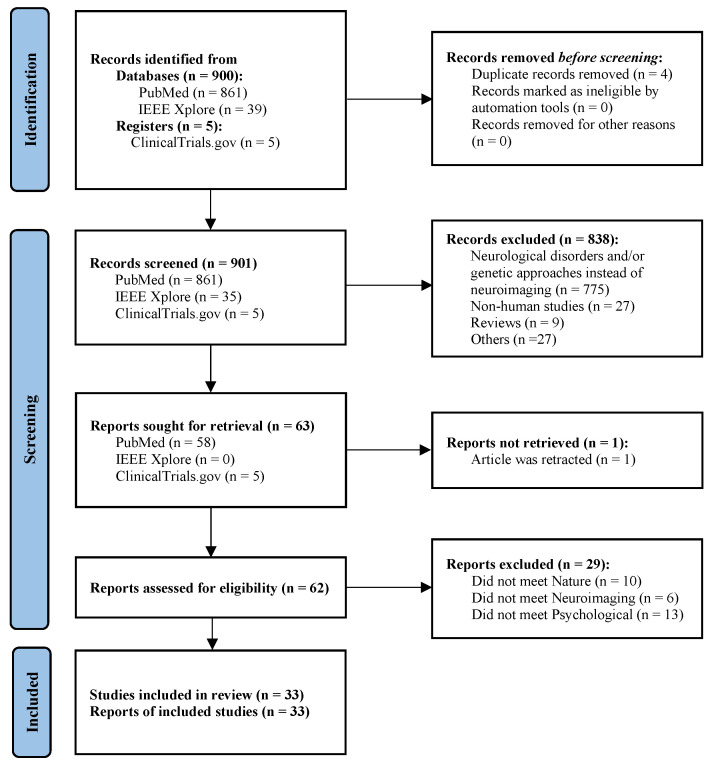
**PRISMA flowchart.** The flowchart follows the three stages of PRISMA study selection: identification, screening, and inclusion, reducing the initial 905 records to the 33 included studies. During title and abstract screening, 838 studies were excluded for focusing on neurological disorders, employing genetic or molecular methods instead of neuroimaging, not involving human participants, including review articles despite keyword filters, or emphasizing alternative approaches such as deep learning-based emotion classification.

PRISMA (Preferred Reporting Items for Systematic Reviews and Meta-Analyses), a systematic review and meta-analysis procedure [31], was used to identify and screen studies as shown in Figure 1. The protocol of the systematic review was registered on PROSPERO, an international systematic review registry, with the registration number CRD420251159732 [32].

### 2.1. Search Strategy and Eligibility Criteria

The search included all studies conducted up to 16 March 2024 and was performed across two bibliographic databases (PubMed and IEEE Xplore) and one clinical trial registry ClinicalTrials.gov). Searches were conducted using the following predefined keywords:(((“EEG” [Title/Abstract] OR “electroencephalogram” [Title/Abstract] OR “fMRI” [Title/Abstract] OR “fNIRS” [Title/Abstract] OR “Functional near-infrared spectroscopy” [Title/Abstract] OR “brain activity” [Title/Abstract]) AND (“health” [Title/Abstract] OR “disease” [Title/Abstract] OR “disorder” [Title/Abstract] OR “brain injury” [Title/Abstract] OR “stress” [Title/Abstract] OR “mood” [Title/Abstract]) AND (“nature” [Title/Abstract] OR “therapeutic garden” [Title/Abstract] OR “urban” [Title/Abstract] OR “natural setting” [Title/Abstract])) NOT (“review” [Publication Type] OR “systematic review” [Publication Type] OR “systematic review” [Title/Abstract])).

For the records identified through ClinicalTrials.gov, only studies with publicly available results at the time of the search were considered eligible. Duplicates were assessed through Zotero [33], and studies that did not meet the following inclusion criteria were excluded:

- **Experimental Task**: Studies had to incorporate some form of nature exposure. This could include visual imagery (images or videos), virtual reality (VR), walking in natural settings, or the use of natural sounds. This exposure could be indoors or outdoors.
- **Neuroimaging Modality**: Exclusively noninvasive neuroimaging modalities were considered, specifically EEG, fMRI, and fNIRS. Studies lacking at least one of these modalities were excluded.
- **Psychological Assessments**: Studies were required to include qualitative or subjective psychological assessments to enable the interpretation of how psychological responses relate to neural activity.

### 2.2. Data Extraction

The following data categories were collected:

(a) **Study Information**
  (i) **Subjects:** The demographic details of participants, including their gender, age groups, location, and the total sample size.
  (ii) **Experimental Population:** The clinical condition of participants, specifying whether they were healthy or diagnosed with a chronic disease.
  (iii) **Experimental Task**
    (i) **Imagery:** Studies using images or videos of nature as a stimulus were categorized as Imagery.
    (ii) **Virtual Reality:** Studies using virtual reality to simulate the effects of nature were classified as VR.
    (iii) **Biophilic:** Tasks involving participants’ physical presence with nature either indoors or outdoors were classified as Biophilic.
    (iv) **Walking:** Studies involving walking in nature were classified separately from Biophilic activities.
    (v) **Sound:** Studies using the sounds of nature as a stimulus were categorized as Sound.
    (vi) **Study Duration:** The number of sessions and the frequency of the experimental procedures.
(b) **Neuroimaging Modality**
  (i) **Modality Type:** The neuroimaging modality used in each study, including cases where multiple modalities were implemented.
  (ii) **Number of Channels:** When applicable, the number of channels used in the modality.
  (iii) **Channel Locations:** For applicable studies, channel locations were noted according to the 10–20 International System. For fMRI studies, the type of magnet implemented was included.
  (iv) **Outcomes:** The neurophysiological outcomes extracted from each study, describing how nature exposure influenced brain activity.
(c) **Psychological Assessments**
  (i) **Assessment Type:** The psychological assessments used in each study, including cases where multiple assessments were implemented.
  (ii) **Outcomes:** The results from the psychological assessments, focusing on emotional changes due to nature exposure.

### 2.3. Assessment of Methodological Quality

The Quality Assessment Tool for Quantitative Studies, based on the Effective Public Health Practice Project (EPHPP) guidelines, was used to evaluate the methodological quality of each identified study [34]. This tool assesses several domains, including selection bias, study design, confounders, blinding, data collection methods, and withdrawals/dropouts. Each domain is rated as strong, moderate, or weak and an overall rating is then assigned to each study accordingly.

To ensure reliability and minimize bias, the assessment was conducted independently by two reviewers, and any discrepancies were resolved by a third reviewer to reach a final consensus. Inter-rater agreement across reviewers was quantified using Fleiss’ Kappa statistic [35].

### 2.4. Meta-Analyses

Meta-analyses were performed using the Forest Plot, which graphically summarized the consistency and statistical significance of the included studies [36]. Only studies that explicitly defined both control and experimental groups were considered in these analyses. A control group was defined as a group that either (1) did not undergo the same intervention as the experimental group (e.g., participants not exposed to a nature intervention), or (2) differed in terms of their health status (e.g., a healthy cohort serving as a comparison for a clinical group).

Given the diversity of psychological scales used across studies, as some instruments assess negative emotions (e.g., anxiety, stress, and tension) and others assess positive emotions (e.g., mood, relaxation, and self-esteem), the directionality of outcomes was standardized to ensure consistent interpretation. Specifically, the scores from assessments in which lower values indicate improvement (i.e., reductions in negative emotional states) were inverted so that positive Hedge’s g values uniformly reflect beneficial effects of nature exposure.

To provide a more comprehensive synthesis and a clear distinction between the subjective and objective indicators of well-being, two separate meta-analyses were performed: one summarizing psychological outcomes (i.e., changes in emotional states based on the psychological assessments), and another summarizing neuroimaging findings (i.e., neural changes derived from the different modalities).

The main outcome measure displayed in the Forest Plot is the effect size estimated using the Hedge’s g [36]. Hedge’s g is a variation of the standardized mean difference, providing a corrected estimation suitable for small sample sizes, thereby offering a more accurate estimation than Cohen’s d when dealing with groups of fewer than 20 participants. Hedge’s g was computed with Equation (1), using the mean difference between the experimental (${\bar{x}}_{E}$) and control (${\bar{x}}_{C}$) groups, standardized by the pooled standard deviation ($s_{E}$: experimental; $s_{C}$: control), and corrected with a small sample size bias correction ($j=1-\frac{3}{4(n_{1}+n_{2})-9}$), as described in [37].

(1) $$g=j\times \frac{{\bar{x}}_{E}-{\bar{x}}_{C}}{\sqrt{\frac{s_{E}^{2}\left(n_{1}-1\right)+s_{C}^{2}\left(n_{2}-1\right)}{n_{1}+n_{2}-2}}}$$

In several studies, both pre-test and post-test data are reported for the experimental and control group. Under these conditions, the canonical version of the Hedge’s g estimation is adapted to account for the within-group changes across different time points; see Equation (2).

(2) $$g=j\times \frac{\left({\bar{x}}_{E_{post}}-{\bar{x}}_{E_{pre}}\right)-\left({\bar{x}}_{C_{post}}-{\bar{x}}_{C_{pre}}\right)}{\sqrt{\frac{s_{E_{pre}}^{2}\left(n_{1}-1\right)+s_{E_{post}}^{2}\left(n_{1}-1\right)+s_{C_{pre}}^{2}\left(n_{2}-1\right)+s_{C_{post}}^{2}\left(n_{2}-1\right)}{2(n_{1}+n_{2}-2)}}}$$

Additionally, for certain studies that lacked an explicit reporting of means and standard deviations for each population, data were extracted from the figures in the published articles using WebPlotDigitizer (version 5.2) [38]. In cases where only partial $\eta^{2}$ values were reported, Hedge’s g was estimated using Equation (3) [39]. Once the Hedge’s g values were estimated, the 95% confidence intervals (CI) were obtained.

(3) $$2\times j\times \sqrt{\frac{\eta^{2}}{1-\eta^{2}}}$$

Subsequently, a pooled summary effect was computed using R Studio (version 4.5.1) [40] to evaluate the heterogeneity among studies. Heterogeneity was evaluated using the Cochran’s Q test, complemented by the $I^{2}$ statistic, which quantifies heterogeneity as a percentage and provides a more robust measure of consistency across studies. A random effects model was applied in all meta-analyses, as the included studies varied in terms of participants’ characteristics, the experimental environments, and the psychological assessments. This model assumes that each study estimates a different, yet related, true effect size and incorporates both within-study sampling error and between-study variance to provide a more generalizable estimate of the pooled effect [41]. Lastly, to assess the potential risk of publication bias related to study weighting or sensitivity, Egger’s regression test based on funnel plot asymmetry was performed.

## 3. Results

The systematic search identified a total of 905 studies. During the initial screening of titles and abstracts, 838 studies from the bibliographic databases were excluded for the following reasons: they focused on neurological disorders and/or employed genetic or molecular methodologies instead of neuroimaging techniques; they did not involve human participants; they included review articles despite the application of keyword filters; or they emphasized alternative approaches, such as deep learning-based emotion classification. Study selection and data extraction were conducted independently by two reviewers, with any discrepancies resolved by a third reviewer to ensure consistency and minimize selection bias. The full texts of the remaining 62 studies were then screened and 33 studies remained according to the inclusion criteria; see Figure 1.

### 3.1. Quality Assessment

The methodological quality of each study was assessed using three categories: strong, moderate, and weak. Most of the studies were identified as weak, with three moderate-quality studies and one strong-quality study; see Table 1.

The inter-rater agreement for the methodological quality assessment was assessed using a subset of 24 studies from the 33 identified, as independent ratings from all three reviewers were available at the initial screening stage. The remaining nine [44,45,56,61,63,67,68,70,71] studies were identified at a later stage of the review process and were assessed by a single reviewer; therefore, they were not included in the agreement analysis.

Across the 24 jointly reviewed studies, agreement beyond chance was poor (κ = −0.07; SE = 0.09), with an observed agreement of 37.50% and an expected agreement of 41.80%. The 95% confidence interval ranged from −0.24 to 0.09, and the level of agreement was not statistically significant (z = −0.85; *p*> 0.05). These results indicate substantial variability in independent quality ratings prior to consensus adjudication.

A total of four studies were not rated as methodologically weak [51,56,58,61]. In each of these studies, EEG was implemented as their neuroimaging modality. Two studies were conducted in VR settings with participants diagnosed with generalized anxiety disorder (GAD, [51]) and cancer [61], while the remaining two were conducted in biophilic settings with healthy participants [56,58].

Although none of these studies implemented State-Trait Anxiety Inventory (STAI) or Profile of Mood States (POMS) (the most commonly used psychological assessments across studies), they reported outcomes consistent with the general findings of this review, including reductions in negative emotions and/or increases in positive emotions following nature exposure.

Of these four higher-quality studies, three were included in the meta-analyses presented in Section 3.7. Ref. [56] was not included, as it did not clearly define both the experimental and control groups. Section 3.7 highlights in more detail the impact that these studies have over the general outcome of this review.

### 3.2. Populations of Interest

#### 3.2.1. Gender Demographics

A total of 2101 participants were identified among the studies analyzed, where 1131 (53.83%) participants identified as female, 919 (43.74%) as male, 50 (2.38%) did not provide sex information, and 1 (0.05%) identified as other.

#### 3.2.2. Geographic Distribution of Participants

Participants were mainly distributed among different populations in Asia (84.63% across twenty-three studies; *n* = 1778 participants), Europe (10.61% across six studies; *n* = 223), United States (3.14% across three studies; *n* = 66), and Australia (1.62% in one study; *n* = 34).

#### 3.2.3. Age Group Distribution

**Figure 2:**
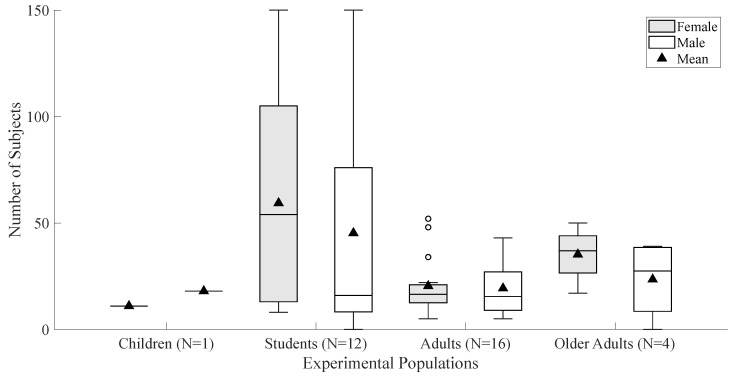
Comparative distribution analysis for the total number of participants across each study. The ‘Δ’ symbol represents the mean, while the line indicates the median. The number of studies that included each population is shown in parentheses next to each group label. The circles in the female adults group indicate individual studies with participant counts substantially higher than those observed in the rest of the studies within that group [47,60,69].

University students comprised a substantial portion of the data, with 1197 (56.97%) participants from 12 of the 33 studies. A single study involved 29 (1.40%) children between ages of seven and thirteen [58], and four studies specifically examined older populations [24,51,56,57], totaling 239 participants (11.40%). The remaining studies recruited adult participants, totaling 636 (30.30%). Figure 2 presents a summary of the participants’ distribution, categorized by both sex and age.

#### 3.2.4. Participant Health Characteristics

The majority of studies focused on healthy participants, with 26 studies (78.80%) comprised exclusively of this population. In total, 1466 (69.80%) participants were categorized as healthy.

In contrast, only six studies included clinical populations incorporating at least one of the following: depression [54,64], GAD [48,51,54,64], hypertension [50,60] and cancer [61]. Anxiety was the condition investigated the most, with four studies examining it; see Table 1. All the studies involving clinical populations consisted of young university-aged adults (mean age: 20.28 years in two studies [54,64]), adults (mean age: 48.05 years in three studies [48,60,61]), and older adults (mean age: 69.16 years across two studies [50,51]).

Notably, only one study included both healthy and clinical (depression) participants within the same investigation [47].

### 3.3. Experimental Tasks

**Figure 3:**
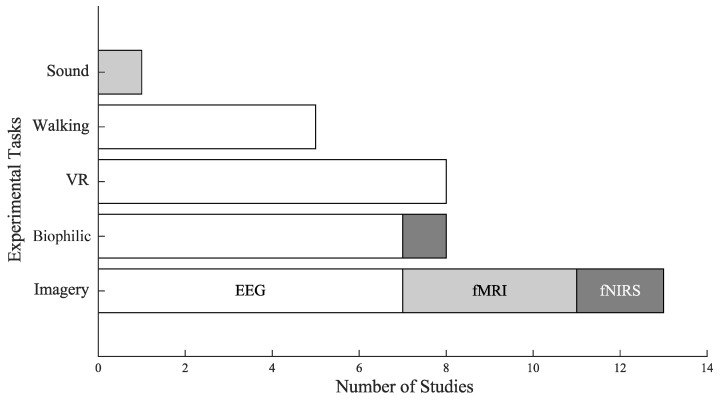
Experimental tasks identified within each of the neuroimaging modalities. The number of measurements totals to 35, larger than the total number of studies (33), due to two studies investigating multiple modalities or experimental tasks.

The experimental tasks were classified into five categories; see Figure 3.

(i) Imagery: The most popular task across the studies (*n* = 13) consistently demonstrated significant effects of nature on well-being. Eight studies reported increases in positive emotions [29,30,46,52,57,59,63,66], while seven observed decreases in negative emotions [29,43,46,52,55,57,63]. Only three studies reported no significant psychological changes following imagery tasks [44,62]. Notably, four of the five studies using fMRI implemented imagery tasks. These findings suggest that imagery is a robust and flexible paradigm for eliciting affective benefits, particularly in controlled laboratory settings.
(ii) Virtual Reality: Eight studies implemented VR-based exposure to nature, all of which used EEG as their neuroimaging modality. Neurophysiological outcomes were primarily derived from changes in EEG frequency band power, and only one study extended to functional connectivity (FC). Six studies reported decreases in negative emotions [49,51,54,61,64,69], and five reported increases in positive emotions [49,51,54,61,69], while two reported a nonsignificant effect psychologically [48,62].
(iii) Biophilic: This task involved interaction with natural environments indoors or outdoors, and the results were assessed primarily using EEG, with one study combining it with fNIRS. Most biophilic studies reported favorable psychological outcomes, with five reporting increases in positive emotions [47,56,58,67,70], and six observed decreases in negative emotions [50,56,58,60,67,70]. Only one study reported no significant change in emotions [53].
(iv) Walking: Studies examined the combined effects of physical activity and exposure to natural environments, with EEG band power as the primary neurophysiological outcome. Five studies reported an increase in positive emotions [28,56,65,68,71], and four reported decreases in negative emotions [28,56,68,71].
(v) Sound: Only one study implemented auditory exposure to natural sounds as the experimental condition, assessed using fMRI [45]. No significant psychological effects were reported. However, the study identified significant neurophysiological changes in terms of increases in functional connectivity and reduced brain entropy (BEN).

### 3.4. Neuroimaging Modalities

Three distinct neuroimaging modalities were used in the studies: EEG (*n* = 27), fMRI (*n* = 5), and fNIRS (*n* = 3), with a single study conducted with both EEG and fNIRS [47].

#### 3.4.1. EEG-Based Studies

EEG was the most widely used neuroimaging modality across the studies reviewed. Neural outcomes were primarily quantified using spectral power analyses across individual frequency bands—delta (0.5–4 Hz), theta (4–8 Hz), alpha (8–13 Hz), beta (14–30 Hz), and gamma (30–40 Hz). A smaller subset of studies implemented alternative metrics, such as Frontal Alpha Asymmetry (FAA), functional connectivity, Attention Quotient (ATQ), and Anti-Stress Quotient (ASQ).

Among the frequency bands examined, alpha band activity emerged as the most reliable and consistent neurophysiological marker of nature exposure. The majority of studies reported increases in alpha power following exposure to natural stimuli, regardless of the type of experimental task implemented. Only two studies identified a decrease in alpha power [56,66].

Modulations within the beta frequency range were the second most commonly reported and exhibited greater variability. Notably, significant beta power modulation was observed predominately in studies involving clinical populations with hypertension [50], anxiety [54], or depression [64]. This pattern suggests that beta oscillations may be particularly sensitive to baseline affective or physiological dysregulation.

The theta range was examined less frequently and was particularly restricted to studies involving healthy adult and student populations. Within these studies, theta power tended to show increases following nature exposure, particularly in experimental paradigms involving immersion or movement, such as walking and biophilic exposure.

Low-frequency oscillations in delta were reported in a limited number of studies. Two investigations identified significant increases in delta power in healthy participants following indirect exposure to nature via imagery [66] and VR [69], suggesting a potential association with deeper restorative or altered states during simulated nature exposure. Lastly, gamma waves appeared in three studies focused on student populations, two of which reported increases [63,70] and one a decrease [66].

Beyond spectral analyses, only one EEG study reported significant changes in FAA [53]. This study found higher FAA scores in urban green spaces (urban park and neighborhood green areas) compared to a busy urban street, in an adult population. Interestingly, it did not identify any significant psychological changes across spaces.

Additionally, a single longitudinal study focusing on children examined EEG-derived ATQ and ASQ metrics [58]. This study reported increases in ATQ values across both hemispheres and increases in ASQ restricted to the left hemisphere, suggesting developmental or hemispheric specificity in attentional and stress-related neural responses to nature exposure.

Finally, seven EEG-based studies reported no significant neurophysiological differences following exposure to natural stimuli, highlighting the ongoing variability in experimental design, population characteristics, and analytic approaches within the current literature [28,47,48,49,52,59,71].

#### 3.4.2. fMRI-Based Studies

Five studies implemented fMRI as their neuroimaging modality including only healthy adults [29,42,43,44,45]. All experiments were performed on 3.0T systems, using scanners manufactured by Siemens (Skyra, Prisma, and Tim Trio) and Philips (Achieva). Each study implemented a single experimental session, with the exception of [43], which conducted two sessions. Neuro-anatomical identification and functional connectivity analyses were conducted implementing established parcellations, such as the Automated Anatomical Labeling (AAL) [72], the seven functional networks by Yeo [73], and the one defined by Gordon and colleagues [74]. Notably, the BEN metric was only implemented in [45].

Neurophysiological findings demonstrated variable correspondence with psychological outcomes. Ref. [42] did not identify significant psychological changes after the nature stimulus, and although they identified that several regions of the brain activated, including the Cerebellar Tonsil, Tuber Gray Matter, Pyramidal Gray Matter, Precentral Gyrus (PCG), Superior Temporal Gyrus (STG), and Insular Cortex, these patterns did not translate into measurable emotional affects. However, the STG has been shown to have a significant change in activation along the ventral Posterior Cingulate Cortex (vPCC), accompanied by reductions in negative emotions on the PSS and the Visual Analog Scale (VAS), indicating a direct alignment between the modulation of self-referential and auditory–integrative regions and improved emotional state [43]. Additionally, Ref. [44] reported no significant psychological outcomes and no difference in brain regions based on the AAL atlas. However, the Yeo’s parcellations showed increased functional connectivity between the Default Mode Network (DMN) and the Dorsal Attention Network (DAN), between the DMN and the Somatomotor Network, and between the DAN and the Ventral Attention Network.

In contrast, clear psychological improvements, reflected in reduced perceived stress and increased mindfulness, were associated with strong activation in the Middle Occipital Cortex (MOC), Supplementary Motor Area (SMA), pre-SMA, Premotor Cortex (PMC), indicating that engagement of these systems may underpin the emotional benefits of exposure to nature stimuli [29]. Lastly, the only study focused on sound [45] identified reduced BEN in a cluster comprising the Posterior Cingulate Gyrus, Cuneus (Cu), pre-Cu, and Occipital lobe/Calcarine. While functional connectivity in the AAL atlas also showed no significant differences, the Gordon parcellations revealed high functional connectivity within Auditory, Cinguloopercular, and Somatomotor (hand and mouth) networks during exposure to nature sounds.

#### 3.4.3. fNIRS-Based Studies

This modality was the least represented among the modalities, with only three studies implementing fNIRS [30,46,47], one of which combined it with EEG [47]. All studies involved healthy participants, although one also included individuals with depression. Each study consisted of single-session protocols. This modality was used to measure cortical hemodynamic activity through changes in the concentration of oxyhemoglobin (O_2_Hb), where increases in O_2_Hb correspond to enhanced neural activity and decreases indicate physiological relaxation connected to reduced brain activity.

Ref. [46] focused on the left and right prefrontal cortex (PFC) and reported decreases in O_2_Hb along with reductions in negative emotions based on STAI and POMS assessments. This parallel pattern suggests a clear relaxation response during exposure to nature scenes. Similarly, Ref. [30] focused in frontal areas in both hemispheres, including the Dorsolateral Prefrontal Cortex (RdlPFC, and LdlPFC) and the Orbitofrontal Cortex (ROFC, and LOFC). However, the study identified a significant decrease in O_2_Hb only in the ROFC, which was associated with increases in positive emotions according to VAS ratings.

Finally, Ref. [47] implemented a broader frontal–occipital configuration in a natural outdoor environment and found no statistically significant hemodynamic effects across sites. Nevertheless, psychological outcomes reflected a reduction in negative emotions.

### 3.5. Other Physiological Measurements

In addition to the neuroimaging modalities, several studies incorporated complementary physiological measurements. Among these, heart rate (HR) was documented in nine studies, whereas heart rate variability (HRV) was measured in four. Only two studies reported both metrics together, each showing an increase in HR. However, only one study observed an increase in HRV [65], whereas the other reported no significant differences [28]. As part of the studies that only included the HR, interestingly, four reported no significant differences [30,46,67,70]. For those with significant findings, Ref. [51] observed an increase, Ref. [52] reported both an increase and a decrease depending on task duration, and Ref. [55] reported a decrease. The results from studies focusing exclusively on HRV were similarly heterogeneous. One study reported a decrease in HRV [57], while the other presented no significant differences [56].

Blood pressure (BP) was included in seven articles and demonstrated great consistency across findings. Five studies reported reductions in BP associated with nature exposure [50,52,55,67,70], while two studies found no significant differences [46,61].

Galvanic Skin Response, an index of electrodermal activity, was assessed in three studies. In these studies, the conclusions were inconsistent: one study found an increase [52], another a decrease [57], and a third reported no significant change with nature [28]. Finally, one study also looked at bioimpedance, where impedance and reactance were reported to increase with nature exposure [56].

### 3.6. Psychological Assessments

#### 3.6.1. Most Common Psychological Assessments

**Figure 4:**
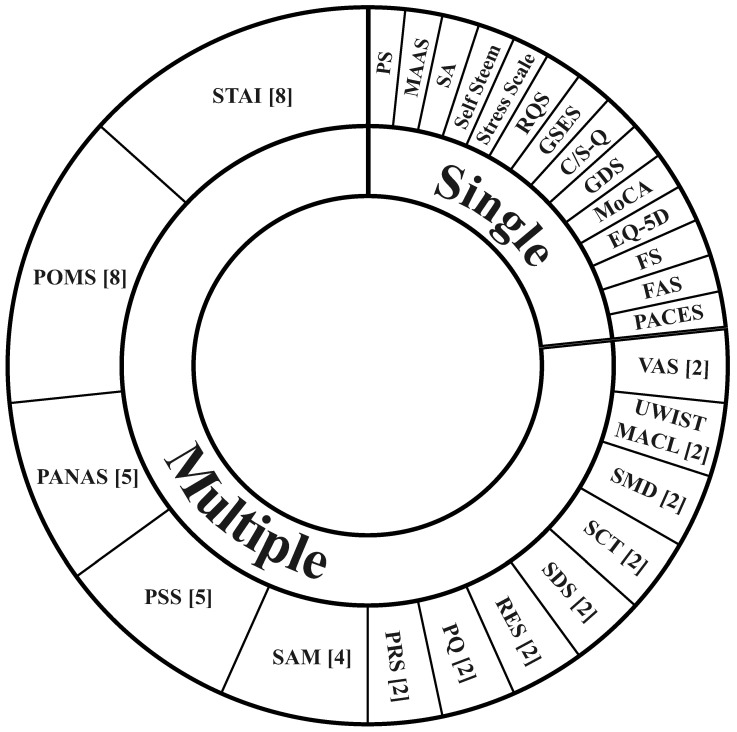
Psychological assessments used across the studies, organized by their number of appearances. The number of studies in which each assessment appears is indicated in brackets.

Across the reviewed studies, a total of 27 different psychological assessments were used to evaluate the subjective effects of nature exposure. Despite the diversity, a small subset of assessments accounted for a substantial proportion of studies; see Figure 4. Most of the assessments relied on Likert-type response formats, typically comprising four- or five-point scales, enabling standardized quantitative evaluation across studies. The STAI [75] and POMS [76] were the most frequently used, appearing in eight studies each. In two instances, both assessments were implemented in parallel [46,55].

STAI was primarily applied in studies with healthy participants (five studies, excluding children), where it consistently evidenced reductions in anxiety and negative emotional states. In addition, STAI was implemented across three clinical populations, including individuals with GAD [48,64], hypertension [50], and depression [64]. Across the clinical contexts, STAI captured improvements characterized by decreasing negative emotions.

In contrast, POMS was used in different formats, including the full-length version [49], the short form [57], and a translated version into mandarin [51,55], highlighting its adaptability across study designs and cultural settings. POMS was implemented across six studies reporting reductions in negative emotions, and four reporting increases in positive mood. Notably, the single study comparing clinical and healthy participants implemented POMS; however, it did not identify any significant changes in mood [47].

Beyond STAI and POMS, several additional assessments were used with moderate frequency, most notably the Positive and Negative Affect Schedule (PANAS; *n* = 5), the Perceived Stress Scale (PSS; *n* = 5), and the Self-Assessment Manikin (SAM; *n* = 4). PANAS was used across both healthy and clinical populations, and consistently captured affective changes following nature exposure, most commonly reductions in negative affect and, in several cases, concurrent increases in positive affect. PSS was employed to assess stress-related outcomes and generally reflected reductions in perceived stress; the sole study reporting no significant change was the one that implemented sound as natural stimuli [45]. SAM was implemented exclusively in healthy participants and uniformly reported enhancements in positive emotional states, particularly in valance and arousal.

#### 3.6.2. Other Psychological Assessments

In addition to the most frequently used assessments, several others were employed with moderate frequency. The Visual Analog Scale, University of Wales Institute of Technology Mood Adjective Check List (UWIST-MACL), Semantic Differential Scale (SMD), Stroop Color Task (SCT), Self-Rating Depression Scale (SDS), Restoration Environment Scale (RES), Presence Questionnaire (PQ), and Perceived Restorativeness Scale (PRS) were each implemented in two studies. Similar to POMS and STAI, many of these scales are measured on a Likert or point-based scoring system. However, the SAM and VAS differ in their approaches. The SAM assesses self-reported emotional states through pictorial and non-verbal representations. Meanwhile, the VAS questionnaire is an uni-dimensional measure where participants mark a point on a continuous line to indicate their state. The SCT differed fundamentally from these self-report measures by providing an objective assessment of attentional and cognitive performance.

Multiple assessment scales were not found in more than one study (see Figure 4). These include the Pleasantness Scale (PS), Mindful Attention Awareness Scale (MAAS), Satisfaction Scale (SA), Self Steem, Stress Scale, Restorative Quality Scale (RQS), Generalized Self-Efficacy Scale (GSES), Comfort and Safety Questionnaire (C/S-Q), Geriatric Depression Scale (GDS), Montreal Cognitive Assessment (MoCA), EuroQol-5 Dimensions (EQ-5D), Feeling Scale (FS), Felt Arousal Scale (FAS), and Physical Activity Enjoyment Scale (PACES).

Several assessments incorporated alternative formats. The GDS focused on items with yes/no responses, while the MoCA screened multiple cognitive domains through task-based evaluation. The EQ-5D combined visual and Likert components, whereas FS relied on semantic differential anchors to capture affective states.

#### 3.6.3. Psychological Assessments Among Populations

Among the Asian studies (*n* = 23), the POMS was the most frequently implemented assessment, as every implementation of POMS occurred in Asian studies, underscoring its high adaptability across different study designs, populations, and cultural contexts. The STAI was the second most common assessment in Asia, appearing in seven studies. The PSS was the only assessment with three separate uses. Several assessments were used twice, namely the SMD, SAM, VAS, PRS, PQ, SCT, SDS, and PANAS. Many were also used once: RES, Stress Scale, Self Steem, RQS, GSES, GDS, MoCA, EQ-5D and the SA.

Among the European studies, there were six different psychological assessments used. The UWIST MACL, PANAS, and PSS were used twice and the STAI, SAM and MAAS were used once. Different scales were used in the three American studies. These scales included the PS, STAI, FS, FAS, and PACES. For the single Australian study, the C/S-Q and the PANAS were used. The single Middle Eastern study used the PRS.

#### 3.6.4. Psychological Outcomes

**Figure 5:**
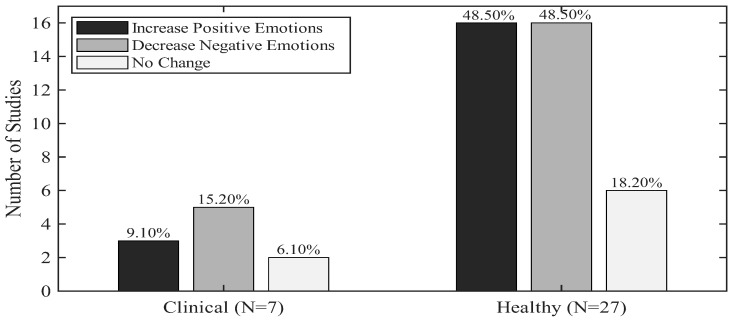
Psychological outcomes in clinical and healthy groups after nature exposure. Due to heterogeneity in psychological assessments, the magnitude of changes in positive or negative emotions were quantified by considering the number of studies reporting beneficial emotional effects. Percentages above bars indicate the proportion of studies relative to the total included in the review (*n* = 33). Totals exceed 33 because one study included both population types.

Given the variety of scales implemented, the findings were categorized into two general outcomes: increase or decrease. This was then applied to the emotional states, focusing on positive and negative emotions directly measured by the scales. It is important to note that some studies analyzed both positive and negative emotions together, while others assessed them separately. Further, the results were analyzed separately for clinical and healthy groups to compare differences in psychological outcomes. Figure 5 shows a summary of the psychological outcomes.

In 48.50% (*n* = 16) of the studies, there was an increase in positive emotions in the healthy groups, which included children [58] and older participants [56,57], while an equal proportion, 48.50% (*n* = 16), reported a decrease in negative emotions, also including children and older participants. In contrast, 18.20% of studies (*n* = 6) reported no significant psychological change in healthy samples.

On the other hand, in clinical populations, reductions in negative emotions were the most frequently observed outcome, reported in five studies, three of which also documented concurrent increases in positive emotions. Studies involving older clinical populations consistently showed decreases in negative emotions, with one study additionally reporting an increase in positive emotional states.

### 3.7. Forest Plot for Meta-Analyses

**Figure 6:**
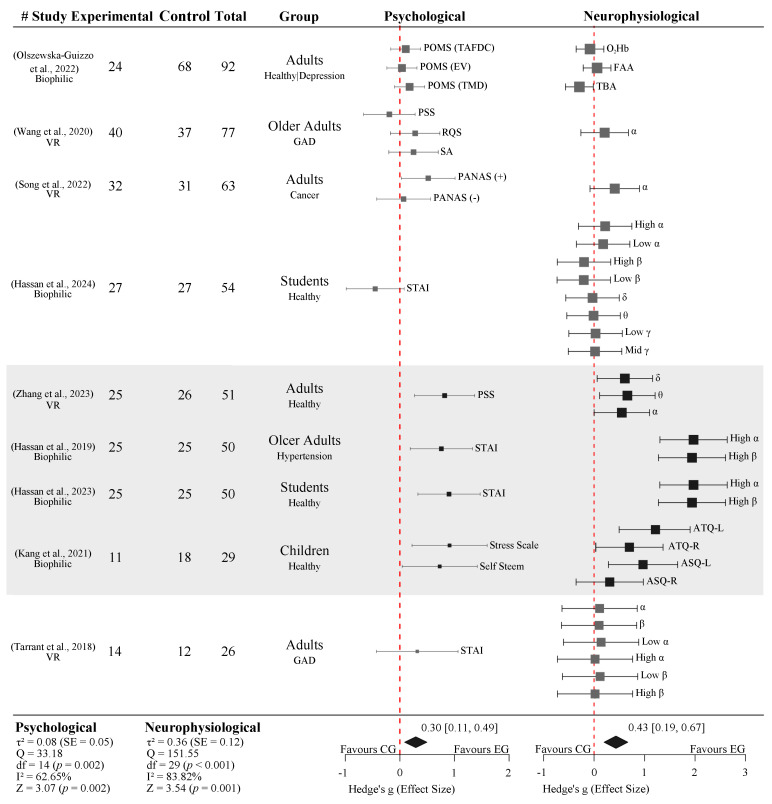
Forest plot summarizing two meta-analyses of nature exposure effects. The red dotted vertical line represents the null effect line, indicating no difference between the experimental and control groups, and serves as the reference dividing effects favoring either group. Results to the left favor the control group, and results to the right favor the experimental group. Marker size reflects study weight. The nine studies included in the analysis are listed in the same order as they appear in the forest plot: [47,48,50,51,58,61,67,69,70].

Figure 6 summarizes the two separate meta-analyses of nine studies that explicitly defined a control and an experimental group, allowing for the direct comparison between nature versus the non-exposure or urban control conditions. One meta-analysis quantifies psychological outcomes, while the other synthesizes neurophysiological effects. Although both meta-analyses are presented within a single figure for integrative interpretation, they represent analytically distinct outcome domains. All included studies implemented EEG as their neuroimaging modality, with one study additionally integrating fNIRS measurements [47]. The analyzed populations encompassed healthy and clinical (depression, gad, cancer, and hypertension) participants, and involved both biophilic and virtual nature exposure.

To ensure comparability across different assessments, all effects were standardized such that positive Hedge’s g values indicated psychological improvement favoring the nature exposure group.

**Figure 7:**
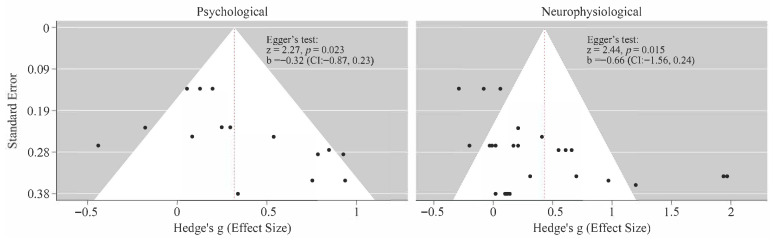
Funnel plots for the meta-analyses of psychological (**left**) and neurophysiological (**right**) outcomes. Each point represents an outcome plotted by Hedge’s g effect size and its standard error. The line in the center indicates the pooled effect estimate from the random effects model.

Under the random effects model, nature exposure was associated with a statistically significant overall positive effect on psychological outcomes (Hedge’s g = 0.30, 95% CI [0.11, 0.49], Z = 3.07, and *p* = 0.002). This effect corresponds to a small-to-moderate improvement in emotional well-being associated with nature exposure. The level of heterogeneity across studies was moderate (I^2^ = 62.65%, Q = 33.18, df = 14, and *p* = 0.002), indicating meaningful variability in effect size across studies, due to the differences in population characteristics, the type of tasks, and the psychological assessments. The funnel plot for psychological outcomes (Figure 7) reports mild asymmetry, meaning that studies with positive results were more common than those with small or null effects. Egger’s regression test suggested possible small study effects (*p* = 0.023). Nevertheless, the degree of asymmetry was limited.

In parallel, the meta-analysis of the neurophysiological outcomes demonstrated a larger pooled effect favoring nature exposure (Hedge’s g = 0.43, 95% CI [0.19, 0.67], Z = 3.54, and *p* < 0.001), followed by substantial heterogeneity (I^2^ = 83.82%, Q = 151.55, df = 29, and *p* < 0.001). Similarly to the psychological outcomes, the funnel plot for neurophysiological outcomes showed asymmetry, supported by Egger’s regression test (*p* = 0.015), suggesting that smaller studies tended to report stronger effects.

## 4. Discussion

This section synthesizes the methodological approaches, outcomes, and gaps identified across the reviewed studies, highlighting key opportunities and providing recommendations to maximize the effectiveness of future research on nature-based interventions and their neurophysiological foundations.

### 4.1. Psychological and Neurophysiological Effects of Nature Exposure Across Populations

Across the reviewed studies, exposure to nature was consistently associated with beneficial psychological outcomes across a wide range of participants, including healthy adults, older individuals, children, and clinical populations. These benefits were primarily expressed as increases in positive emotions, decreases in negative emotions, or a combination of both. The neurophysiological findings generally aligned with these psychological effects, although they showed greater variability due to the type of modality, analytic approach, and experimental design.

The most important aspect of the studies included is that the majority were conducted in Asia, accounting for almost 70% of the studies. This regional dominance shaped several aspects of the literature, including the choice of psychological assessments, experimental tasks, and target populations, limiting the generalizability of the findings and underscoring the need for broader cross-cultural replication.

#### 4.1.1. Differences Between Healthy and Clinical Populations

When comparing between clinical and healthy samples, different patterns emerged. In healthy populations, psychological improvements were equally distributed between decreases in negative emotions and increases in positive emotions. In contrast, studies with clinical populations emphasized reductions in negative emotions, with fewer reports of increases in positive emotions. These differences likely reflect the contrast in emotional processing.

Clinical populations, particularly those with GAD or depression, are characterized by heightened negative affect and attentional biases toward negative stimuli. As a result, decreases in negative emotional states may represent the most clinically meaningful benefit of nature exposure in these groups. Despite the limitation on the number of studies including clinical populations, it is possible to highlight the potential therapeutic relevance of nature-based interventions for emotional regulation and well-being.

#### 4.1.2. Variation Across Populations, Regions, and Age Groups

Variations in psychological and neurophysiological outcomes were evident across regions and age groups. STAI emerged as the most widely used assessment across regions, reflecting its strong psychometric properties and simplicity. In contrast, POMS was implemented only in Asian studies, highlighting its adaptation to different languages and cultural contexts. Similarly, studies conducted in the United Kingdom preferentially employed the UWIST MACL, reflecting the regional origins and established used of this assessment.

Regarding the age-related differences, the single study involving children implemented assessments focused on stress and Self Steem, suggesting that nature exposure in early life stages may primarily support emotional regulation rather than mood enhancement. Student populations were the most common across studies and the positive outcomes may suggest that nature exposure can be effective in modulating short-term affective states, as students are commonly under cognitive demands and stressors related to academic environments.

Among adults, nature exposure was associated with a wide range of affective and stress-related outcomes, reflecting their greater emotional differentiation and self-report capacity. In older adults, psychological benefits mirrored the benefits of clinical populations, as they reported a decrease in negative emotions, and their neural responses in these groups were typically interpreted in relation to reductions in negative emotional states rather than enhancements of positive affect.

Importantly, the interpretation of psychological and neurophysiological responses across different populations and age ranges strengthens the interpretation that nature exposure engages underlying brain mechanisms relevant to emotional regulation. At the same time, the observed variability highlights the importance of considering population- and context-specific factors when interpreting the effects of nature on well-being.

### 4.2. Influence of Experimental Task on Psychological and Neurophysiological Outcomes

The experimental tasks ranged from direct engagement with natural environments to indirect exposure through imagery, virtual reality, or sound-based stimuli.

#### Direct vs. Indirect Nature Exposure

Direct exposure tasks, particularly walking and biophilic activities, produced the most consistent psychological benefits across studies. These tasks also provided a clearer picture of the neurophysiological changes, supporting theoretical frameworks such as ART and SRT, which emphasize multi-sensory engagement and present interaction with natural environments. In contrast, indirect exposure tasks demonstrated psychological benefits but were associated with more variable neurophysiological findings.

Notably, the majority of studies reporting no significant changes after nature exposure implemented indirect tasks, suggesting that the lack of a real nature interaction may attenuate measurable neural responses. Within this group, the single study using sound-based exposure did not report significant psychological outcomes. This was unexpected as prior research has indicated the use of sound as a therapeutic mechanism, as nature sounds have demonstrated effects on reducing stress [77,78]. This indicates that this experimental task should be investigated further to understand the psychological impacts. Consistent with this interpretation, previous work has indicated that direct interactions with nature elicits stronger benefits than indirect forms of exposure [79].

Virtual reality occupied a place between direct and indirect exposure. Psychologically, VR was more associated with the reduction in negative emotions. These finding suggest that VR has the potential to support emotional benefits.

### 4.3. Neurophysiological and Psychological Correlates of Nature Exposure

To complement psychological findings and better understand the mechanisms underlying relaxation, reduced cognitive effort, and stress regulation, the reviewed studies included neurophysiological outcomes using EEG, fNIRS, and fMRI, offering distinct perspectives.

EEG was the most accessible neuroimaging modality due to its popularity across diverse contexts and environments. Across studies, EEG demonstrated a high sensitivity to nature exposure, with significant neural changes reported across most experimental tasks. However, EEG findings were not uniformly consistent, particularly in studies involving walking tasks. In these cases, nonsignificant results may be partly attributable to motion-related artifacts, which are a known challenge in mobile EEG recordings. Importantly, most studies provided limited detail about preprocessing pipelines, artifact rejection strategies, or motion correction procedures, constraining reproducibility and cross-study comparability. Although advanced EEG denoising algorithms capable of addressing movement, muscle, and ocular artifacts are available [80,81], their adoption and reporting remain inconsistent. Addressing these methodological gaps is critical for improving the reliability of EEG-based investigations of nature exposure, particularly in ecologically valid, mobile settings.

fMRI was a distant second-most popular modality, predominantly in Imagery and Sound studies. Due to its stationary and enclosed setup, fMRI is limited to static experimental settings, which may compromise ecological validity when studying nature’s dynamic and immersive effects. Imagery studies found some consistent areas of activation, but not across all of the studies. The single sound-based study showed similar activation in the Somatomotor Network as in Imagery studies, but overall saw activation in different ROIs. These findings highlight fMRI’s limitations for mapping brain networks engaged during real-world natural experiences.

Even though less common than EEG or fMRI, fNIRS studies reported significant effects in imagery-based studies, whereas the results from the single outdoor activity study were insignificant. This may point to the need for more research implementing fNIRS in naturalistic settings to better understand its applicability and how their physiological outcome relates to the psychological aspects.

#### 4.3.1. EEG Metrics

EEG-focused studies provided the most detailed neurophysiological insight into how nature exposure modulates brain activity, mainly through spectral power, asymmetry measures, and functional connectivity.

Among the power bands examined, alpha waves were the most popular due to their association with relaxation. Prior studies have reported a consistent increase in alpha activity during tasks related to relaxation, such as meditation [82], yoga [83], and nature imagery [84]. These results reinforce the notion that nature provides a calming neural environment.

In contrast, findings for beta, theta, delta, and gamma waves presented inconsistencies, reflecting the complexity of interpreting these signals in the context of nature exposure. Beta waves were associated with attention, cognitive demand, stress [66,68], and even drowsiness [50].

Theta wave increases were associated with mindfulness [60], relaxation [62], and attentiveness [63]. Delta wave interpretations indicated disharmony, being attributed to tiredness [70], and attention or concentration [66]. Although delta waves have traditionally been associated with deep meditation or dreamless sleep [85], recent evidence indicates that they also play a role in encoding movement intention through fluctuations in the amplitude of slow cortical potentials [86,87].

Lastly, gamma waves also reported consistency on studies linking them to relaxation [62]. This highlights gamma waves as a potential reliable marker of neural benefits of nature exposure. However, further research is necessary to validate this hypothesis.

Beyond spectral power, FAA provided a complementary physiological index of the affective state. Notably, the only study reporting significant changes in FAA implemented a biophilic experimental task [53]. Importantly, this neurophysiological outcome was not accompanied by significant changes in self-reported psychological outcomes, highlighting a dissociation between neural and subjective measures. These observations suggest that the relevance of FAA remains uncertain and more research with this is needed as it is defined as an objective measure of mood status [53].

Lastly, a single study examined EEG-derived functional connectivity during a VR task in healthy participants [69]. This study suggested that functional connectivity can be a good marker of decreasing negative emotions after nature exposure. However, this metric needs more research and, also, exploration in real-world environments.

#### 4.3.2. fMRI and fNIRS Metrics

Although fMRI and fNIRS were implemented far less frequently compared to EEG, these neuroimaging modalities provided complementary insights into the neural mechanisms underlying the psychological benefits of nature exposure.

As for fMRI studies, they predominated in imagery- and sound-based tasks, reflecting the limitation in keeping participants in stationary and enclosed environments. Across these studies, neurophysiological outcomes varied, and patterns of activation were observed across distributed regions that form part of broader functional networks, such as the DMN and the Somatomotor Network. The DMN is associated with mindfulness and mind wandering [88], and its activation after nature exposure suggests potential restorative effects on attention and cognition [89]. These findings are consistent with ART, which proposes that natural environments can help restore cognitive resources [4]. Additionally, prior research has demonstrated that nature enhances attention and cognition [90]. In contrast, the Somatomotor Network, while primarily linked to motor control, is also implicated in spatial cognitive operations [91]. Its activation during visual exposure to nature suggests that this network may engage in processing spatial representations of environmental stimuli, further emphasizing the cognitive benefits of natural environments.

Similar to fMRI studies, those implementing fNIRS targeted different ROIs in the brain, making any direct comparisons of outcomes challenging. Nevertheless, their findings presented significant importance, as they align with prior research demonstrating that nature exposure causes a decrease in O_2_Hb levels in the orbitofrontal and prefrontal cortices, while also promoting psychological relaxation [92]. The prefrontal cortex, critical for executive function [93], reveals a decrease in O_2_Hb levels after nature exposure, which indicates a reduction in neural activity and strengthened relaxation [92]. Notably, the fNIRS modality was implemented in Imagery studies, and was integrated with EEG in a study related to Biophilic. These findings demonstrate the high versatility and adaptability to different tasks and environments compared to more restrictive neuroimaging modalities, such as fMRI.

### 4.4. Nature Exposure Within the Neural Exposome Framework

The outcomes of this review and meta-analysis can be interpreted within the neural exposome framework, which emphasizes the importance of nature exposure on the brain’s health across the lifespan. From this perspective, nature exposure may act as a beneficial environmental factor that supports enhanced neural function and psychological well-being throughout an individual’s life.

The meta-analyses demonstrated that exposure to nature was correlated to improvements in psychological outcomes as well as measurable changes in neurophysiological signals. Neurophysiological measures often showed stronger effects than self-reported psychological measures, suggesting that changes in brain activity may be detected even without clear subjective reports. The results varied across studies, consistent with the neural exposome framework, which posits that environmental impacts on the brain depend on health status, type of exposure, and outcome measures.

### 4.5. Limitations and Opportunities

This systematic review presents several limitations and opportunities that should be considered. First, although the search strategy included two bibliographic databases (PubMed and IEEE Xplore) and one clinical registry (ClinicalTrials.gov), all eligible studies were ultimately identified though PubMed. While PubMed is a valuable resource for systematic reviews, it may not capture all relevant research, particularly unpublished studies, conference proceedings, dissertations or studies not indexed in English. Future reviews should incorporate other databases and expand the range of key terms to ensure a more comprehensive identification of potential studies. Nevertheless, given the limited number of eligible studies and the generally low methodological quality of the included publications, it is unlikely that additional database coverage would substantially alter the overall conclusions of this review.

Second, the main focus of this review was to identify the tasks, neurophysiological and psychological metrics, and outcomes of the nature-based interventions, rather than the methodologies implemented for preprocessing neural data. This highlights an important limitation, as the quality of the neural data depends on the effective management of artifacts during preprocessing [94,95]. Many of the studies included in this review did not adequately report on their approaches to cleaning EEG or fNIRS signals, which could explain some of the inconsistencies or null findings. Future studies should include detailed information about preprocessing steps to remove artifacts from the recordings.

Third, the quality of the studies was generally weak (see Table 1), which may affect the reliability of the results. To mitigate this, future studies should prioritize enhancing the methodological quality to improve internal validity and minimize scientific errors. Moreover, EEG studies should expand their scope to include additional and more comprehensive metrics such as functional connectivity, to better characterize large-scale brain networks. Importantly, this will require the use of higher-density EEG systems with an increased number of channels to improve spatial resolution, as well as a more explicit linkage between psychological assessments and neuroimaging outcomes.

Fourth, future studies with experimental tasks requiring physical presence in nature should also include a broader range of environments and climate conditions as well as longitudinal data to account for changes in environmental conditions. Importantly, it is feasible to implement a standardized characterization of the environments in which experiments are conducted based on quantitative indices such as population density, vegetation density, built environment intensity, traffic load, and acoustic characteristics. This approach would harmonize differentiators between urban and nature environments across studies. Currently, nature exposure can be conceptually organized along a spectrum, from imagined exposure, to virtual environments, to immersive real-world settings. A similar spectrum could, in principle, be applied to urban environments; however, most studies do not report objective environmental metrics, limiting the ability to systematically characterize urbanicity along a continuous gradient. However, a careful evaluation of technology should be considered, as functionality and performance can vary significantly depending on the environmental conditions. Exploring different environments aligns with the research needs identified by the U.S. National Institute of Environmental Health Sciences, which emphasizes the need for a better understanding of how psychological stress can interact with different environmental exposures [96].

Fifth, most of the studies (70%) identified in this review were conducted in Asia, which limits the applicability of the findings to the Western population and, in particular, the United States. Cultural and environmental aspects should be considered in future studies, which can also be plausibly done through collaborative trans-national and multi-institutional efforts.

Sixth, the lack of standardized event labeling, data harmonization, and open data practices represents a major barrier to the reproducibility of studies of human–nature interactions. In real-world settings, the complexity and variability of interactions between participants and natural environments are often insufficiently characterized, making it difficult to accurately represent experimental conditions and compare outcomes across studies. A conceptual framework proposed in [97] offers a valuable foundation for addressing this challenge by deconstructing human–nature interactions into five key dimensions:

- Immediateness: Subjects’ physical proximity with respect to nature (e.g., walking in green areas, animal interaction, and touching flowers). This dimension has two subcategories: direct (physical and close interaction with nature) and indirect (no physical presence in the wildlife’s environment), where both categories consider the spatial factors surrounding the subject.
- Consciousness: The level of awareness present during the interaction (e.g., acknowledgment of an animal’s presence and observation of wildlife), branching into conscious and subconscious (e.g., background noise and passive observation).
- Intentionality: This relates heavily with the subject’s level of awareness (i.e., consciousness), described as the deliberateness of the interaction taking place; it is distinguished as intentional (chosen) and less intentional (incidental), where it can be determined as a targeted effort (e.g., feeding animals) or a byproduct of other activities (e.g., walking in the forest, rock climbing, spontaneous encounters).
- Degree of human mediation: The extent to which the setting includes anthropogenic alterations, ranging across two different environments, human-mediated (e.g., city gardens, zoos, and nature reserves) and natural (e.g., remote locations with wildlife)
- Direction of outcomes: Positive and negative results from subjects’ interactions with the environment.

This structured characterization of interactions would improve interpretability, enhance cross-study comparability, and support more meaningful correlations between psychological assessments and underlying neurophysiological outcomes.

Beyond event definition, the standards and harmonization of data acquired in real-world settings should be a priority to promote comparisons, data sharing, and the application of Artificial Intelligence (AI) methods to such datasets.

Finally, it is clear that investments in improving the portability, usability, interoperability, form factor, and reliability of EEG and fNIRS systems should also be prioritized to improve data quality and consistency, while allowing for the aggregation of environmental data such as climate, GPS, and other environmental data such as odor.

Supporting the theorized connection between nature and human well-being, the reviewed studies found that overall, exposure to nature positively impacted participants with increases in positive emotions and/or decreases in negative emotions. However, quantifying this impact was more consistent psychologically than neurophysiologically.

## 5. Conclusions

The studies included in this review demonstrate a growing interest in understanding the impact of nature on the brain’s networks and well-being. While the psychological benefits of nature have been studied for decades, the incorporation of neuroimaging techniques is relatively recent. Across studies, nature exposure was generally associated with favorable psychological outcomes; however, neurophysiological findings were more variable, exhibiting differences across populations, experimental tasks, and study quality.

Within the neural exposome framework, these findings suggest that nature exposure may represent a factor capable of influencing brain processes related to emotional regulation. The meta-analyses results provide quantitative support for this interpretation, showing significant pooled effects for both psychological and neurophysiological findings, while also underscoring substantial heterogeneity.

Overall, EEG was the most popular neuroimaging modality and frequently indicated positive effects of nature exposure through alpha band power analysis consistent with increased positive emotions and/or a decrease in negative emotions. In contrast, fMRI and fNIRS findings offered complementary insights into brain-network-level processes but presented limitations regarding the amount of studies and ecological validity. Additionally, most fMRI and fNIRS studies lacked appropriate control groups or did not report statistical information required for inclusion in quantitative analysis, limiting their contribution to the meta-analytic inference. Taken together, these observations point to the promising correlation between neurophysiological and psychological outcomes highlighting the need for more rigorous and standardized research in the field.

Significantly, although peripheral physiological outcomes may be associated with autonomic or cardiovascular responses associated with nature exposure, the variability observed across studies suggests that such measures alone are insufficient to explain the mechanisms responsible for these effects. When it comes to mechanistic inference, peripheral indicators lack the spatial and functional specificity required to link environmental exposure directly to brain network modulation. This reinforces the need for neuroimaging approaches.

From a science–policy perspective, these findings have important implications. If the benefits of nature exposure want to be incorporated into public health, urban planning, or environmental policy frameworks, stronger foundations are needed. Future studies should prioritize methodological standardization, the inclusion of diverse and adequately represented populations, multimodal and longitudinal approaches. The use of MoBI technology offers a particular promise to allow the study of mechanisms underlying the neural exposome in real-world settings.

## Figures and Tables

**Table 1:** Studies assessed in this research.

Study	Task	Sessions	Groups	Condition	Modality	Psychological Assessment	# Ch or Magnet	Ch locs or ROIs	Psychological Outcomes	Neuroimaging Outcomes	Quality Assessment
[42]	Imagery	1	10 A (5F, 5M)	Healthy	fMRI	PS	Siemens Skyra 3.0T	–	–	–	W
[43]	Imagery	2	44 A (22F, 22M)	Healthy	fMRI	PSS VAS	Philips Achieva 3.0T	dPCC|vPCC MD|STG|SPL mFG|PCG|Cu	$E_{-}^{↓}$	vPCC^↑^, STG^↑^	W
[44]	Imagery	1	24 A (16F, 8M)	Healthy	fMRI	SAM	Siemens Prisma 3.0T	AAL & Yeo atlas	–	FC(Yeo)^↑^	W
[29]	Imagery	1	49 A (11F, 38M)	Healthy	fMRI	PSS MAAS	Siemens Tim Trio 3.0T	PMC|pre-SMA SMA|MOC	PSS: $E_{-}^{↓}$ MAAS: $E_{+}^{↑}$	MOC, SMA, PMC, pre-SMA^↑^	W
[45]	Sound	1	35 A (12F, 23M)	Healthy	fMRI	PANAS PSS	Siemens Tim Trio 3.0T	Gordon & AAL atlas	–	BEN^↓^ FC(Gordon)^↑^	W
[46]	Imagery	1	18 A (9F, 9M)	Healthy	fNIRS	STAI POMS	–	PFC	$E_{-}^{↓}$	O_2_Hb^↓^	W
[30]	Imagery	1	25 S (9F, 16M)	Healthy	fNIRS	VAS	52	RdlPFC (13|23|24) LdlPFC (18|28|29) ROFC (45|46|47) LOFC (48|49|50)	$E_{+}^{↑}$	O_2_Hb[ROFC]^↓^	W
[47]	Biophilic	1	92 A (52F, 40M)	Healthy Depression	fNIRS EEG	POMS	8 16	BAs: 8|9|10|17 18|44|45|46 AFp1|AFp2|F7|F8 AFF5h|AFF6h|FT7|T7 FCC3h|FCC4h|FT8|T8 P3|P4|Ol1h|Ol2h	–	–	W
[48]	VR	1	26 A (20F, 6M)	GAD	EEG	STAI	19	Fp1|Fp2|F3|F4|F7|F8 Fz|C3|C4|Cz|P3|P4|Pz T3|T4|T5|T6|O1|O2	–	–	W
[28]	Walking	1	34 A (20F, 14M)	Healthy	EEG	PANAS	14	AF3|AF4|F3|F4 F7|F8|FC5|FC6 T7|T8|P7|P8 O1|O2	$E_{+}^{↑}$ & $E_{-}^{↓}$	–	W
[49]	VR	1	120 S (62F, 58M)	Healthy	EEG	POMS SCT	1	Fp1	POMS: $E_{-}^{↓}$	–	W
[50]	Biophilic	1	50 O F	Hypertension	EEG	STAI	1	Fp1	$E_{-}^{↓}$	[α, β${]}_{High}^{↑}$	W
[51]	VR	1	77 O (38F, 39M)	GAD	EEG	RQS, SA PSS	4	Fp1|Fp2 T3|T4	RQS^↑^, SA^↑^ PSS: $E_{-}^{↓}$	–	M
[52]	Imagery	1	180 S (90F, 90M)	Healthy	EEG	POMS	2	Fp1|Fp2	$E_{-}^{↓}$ & $E_{+}^{↑}$	–	W
[53]	Biophilic	1	22 A (13F, 9M)	Healthy	EEG	POMS	16	Fp1|Fp2|F5 F6|F7|F8	–	FAA^↑^	W
[54]	VR	1	189 S (113F, 76M)	Anxiety Depression	EEG	PANAS, PQ GSES, RES	2	Fp1|Fp2	PRS: $E_{+}^{↑}$ GSES: $E_{+}^{↑}$ PANAS: $E_{-}^{↓}$	$\beta^{↑}$	W
[55]	Imagery	1	300 S (150F, 150M)	Healthy	EEG	POMS STAI	1	Fp1	$E_{-}^{↓}$ & $E_{+}^{↑}$	$\alpha_{High}^{↑}$ & $\beta_{High}^{↓}$	W
[56]	Walking Biophilic	12	74 O (36F, 38M)	Healthy	EEG	MoCA, GDS EQ-5D	2	Fp1|Fp2	Cognition^↑^ Depression^↓^ QoL^↑^	Walking: $\alpha^{↑}$ Biophilic: $\alpha^{↓}$, $\beta^{↑}$	M
[57]	Imagery	1	34 O (17F, 17M)	Healthy	EEG	SMD POMS	14	AF3|AF4|F3|F4 F7|F8|FC5|FC6|T7 T8|P7|P8|O1|O2	SMD: $E_{+}^{↑}$ POMS: $E_{-}^{↓}$ & $E_{+}^{↑}$	$\alpha^{↑}$	W
[58]	Biophilic	8	29 C (11F, 18M)	Healthy	EEG	Stress Scale Self Steem	2	Fp1|Fp2	SES: $E_{+}^{↑}$ SS: $E_{-}^{↓}$	ATQ-L^↑^, ATQ-R^↑^ ASQ-L^↑^	S
[59]	Imagery	2	25 A (14F, 11M)	Healthy	EEG	POMS SAM	16	AFF5h|AFF6h	SAM: $E_{+}^{↑}$	–	W
[60]	Biophilic	1	79 A (48F, 31M)	Healthy	EEG	SAM	16	AFp1|AFp2|AFF5h AFF6h|F7|F8|FT7|FT8 FCC3h|FCC4h|T7 T8|P3|P4|OI1h|OI2h	Arousal^↑^	[θ, α, β${]}_{Median}^{↑}$	W
[61]	VR	3–5	63 A (20F, 43M)	Cancer	EEG	SDS, PRS PANAS	3	Fp1	PANAS: $E_{-}^{↓}$ & $E_{+}^{↑}$	–	S
[62]	VR Imagery	1	31 S (22F, 9M)	Healthy	EEG	STAI UWIST MACL	32	Fp1|Fp2|Fz|F3|F4 F7|F8|Oz|O1|O2	–	[θ, α, γ]^↑^	W
[63]	Imagery	1	30 S (25F, 5M)	Healthy	EEG	UWIST MACL	32	10–20 System	Historic Site: $E_{-}^{↓}$ & $E_{+}^{↑}$	[θ, α]^↑^	W
[64]	VR	1	186 S (110F, 76M)	GAD Depression	EEG	RES, PQ STAI, SDS	2	Fp1|Fp2	SDS & STAI: $E_{-}^{↓}$	$\alpha^{↑}$, $\beta^{↓}$, $\alpha /\beta^{↑}$	W
[65]	Walking	1	30 A (17F, 13M)	Healthy	EEG	FS, FAS PACES	24	F7|F8|P7|P8	Attention^↑^ Enjoyment^↑^ Emotional Awar.^↑^	[θ, α]^↑^	W
[66]	Imagery	1	20 S (10F, 10M)	Healthy	EEG	SAM	64	10–20 System	Valence^↑^ Arousal^↑^ Dominance^↑^	$\delta^{↑}$, [θ, α, β, γ]^↓^	W
[67]	Biophilic	1	50 S	Healthy	EEG	STAI	1	Fp1	$E_{-}^{↓}$	[α, β${]}_{High}^{↑}$	W
[68]	Walking	1	34 A (13F, 20M, 1Other)	Healthy	EEG	C/S-Q PANAS	4	TP9|TP10|AF7|AF8	Comfort^↑^ Safety^↑^ PANAS: $E_{-}^{↓}$	$\theta^{↑}$, $\beta^{↓}$	W
[69]	VR	1	51 A (34F, 17M)	Healthy	EEG	SCT PSS	18	Fp1|Fp2|F7|F3|Fz|F4 F8|T3|T4|C3|Cz|C4 T5|T6|P3|Pz|P4|POz	PSS: $E_{-}^{↓}$	[δ, θ]^↑^ FC^↑^	W
[70]	Biophilic	1	54 S F	Healthy	EEG	STAI SMD	1	Fp1	SMD: $E_{+}^{↑}$ STAI: $E_{-}^{↓}$	–	W
[71]	Walking	1	16 S (8F, 8M)	Healthy	EEG	PRS	1	Fp1	$E_{-}^{↓}$ & $E_{+}^{↑}$	–	W

**Groups:** students (S), adults (A), older adults (O), children (C), female (F), and male (M). **Psychological Outcomes:** $E_{+/-}^{↑↓}$: emotions (E). Type of Emotions: positive (+) or negative (−), increase (↑) or decrease (↓). **Neuroimaging Outcomes:** [Type of metric]^↑↓^: increase (↑) or decrease (↓). **Quality Assessment:** weak (W), moderate (M), or strong (S).

## Data Availability

No new data were created or analyzed in this study. Data sharing is not applicable to this article.

## Abbreviations

The following abbreviations are used in this manuscript:

**Physiological Measures**	
ATQ	Attention Quotient
ASQ	Anti-Stress Quotient
BEN	Brain Entropy
BP	Blood Pressure
EEG	Electroencephalography
FAA	Functional Alpha Asymmetry
FC	Functional Connectivity
fMRI	Functional Magnetic Resonance Imaging
fNIRS	Functional NearInfrared Spectroscopy
HR	Heart Rate
HRV	Heart Rate Variability
O_2_Hb	Oxyhemoglobin
**Psychological Assessments**	
AS	Attention Scale
C/S-Q	Comfort and Safety Questionnaire
EQ-5D	EuroQol-5 Dimensions
FAS	Felt Arousal Scale
FS	Feeling Scale
GDS	Geriatric Depression Scale
GSES	Generalized Self-Efficacy Scale
MAAS	Mindful Attention Awareness Scale
MoCA	Montreal Cognitive Assessment
PACES	Physical Activity Enjoyment Scale
PANAS	Positive and Negative Affect Schedule
POMS	Profile of Mood States
PQ	Presence Questionnaire
PRS	Perceived Restorative Scale
PS	Pleasantness Scale
PSS	Perceived Stress Scale
RES	Restoration Environment Scale
RQS	Restorative Quality Scale
SA	Satisfaction Scale
SAM	Self-Assessment Manikin
SCT	Stroop Color Task
SDS	Self-Rating Depression Scale
SES	Self Esteem Scale
SDS	Semantic Differential Scale
SMS-PA	State Mindfulness Scale for Physical Activity
SS	Stress Scale
STAI	State-Trait anxiety Inventory
UWIST-MACL	University of Wales Institute of Technology Mood Adjective Check List
VAS	Visual Analog Scale
**Regions of Interest**	
AAL	Automated Anatomical Labeling
BA	Brodmann Area
Cu	Cuneus
DAN	Dorsal Attention Network
DMN	Default Mode Network
dPCC	Dorsal Posterior Cingulate
LdlPFC	Left Dorsolateral Prefrontal Cortex
LOFC	Left Orbitofrontal Cortex
MD	Medial Dorsal Nucleus of the Thalamus
mFG	Middle Frontal Gyrus
MOC	Middle Occipital Cortex
Oc	Occipital Lobe
OFC	Orbitofrontal Cortex
PCG	Precentral Gyrus
PFC	Prefrontal Cortex
PMC	Premotor Cortex
RdlPFC	Right Dorsolateral Prefrontal Cortex
ROFC	Right Orbitofrontal Cortex
SMA	Supplementary Motor Area
SPL	Superior Parietal Lobule
STG	Superior Temporal Gyrus
TBG	Tuber Gray Matter
vPCC	Ventral Posterior Cingulate Cortex
**Others**	
ART	Attention Restoration Theory
CI	Confidence Interval
EPHPP	Effective Public Health Practice Project
GAD	Generalized Anxiety Disorder
MoBI	Mobile Brain–Body Imaging
PRISMA	Preferred Reporting Items for Systematic Review and Meta-Analyses
ROIs	Regions of Interest
SE	Standard Error
SRT	Stress Reduction Theory
VR	Virtual Reality
